# Comparison Between Supervised and Unsupervised Classifications of Neuronal Cell Types: A Case Study

**DOI:** 10.1002/dneu.20809

**Published:** 2010-11-30

**Authors:** Luis Guerra, Laura M McGarry, Víctor Robles, Concha Bielza, Pedro Larrañaga, Rafael Yuste

**Affiliations:** 1 Departamento de Inteligencia Artificial, Facultad de Informatica, Universidad Politécnica de MadridSpain; 2 HHMI, Department of Biological Sciences, Columbia UniversityNew York; 3 Departamento de Arquitectura y Tecnología de Sistemas Informáticos, Facultad de Informática, Universidad Politécnica de MadridSpain

**Keywords:** supervised, classification, clustering, pyramidal cell, interneuron

## Abstract

In the study of neural circuits, it becomes essential to discern the different neuronal cell types that build the circuit. Traditionally, neuronal cell types have been classified using qualitative descriptors. More recently, several attempts have been made to classify neurons quantitatively, using unsupervised clustering methods. While useful, these algorithms do not take advantage of previous information known to the investigator, which could improve the classification task. For neocortical GABAergic interneurons, the problem to discern among different cell types is particularly difficult and better methods are needed to perform objective classifications. Here we explore the use of supervised classification algorithms to classify neurons based on their morphological features, using a database of 128 pyramidal cells and 199 interneurons from mouse neocortex. To evaluate the performance of different algorithms we used, as a “benchmark,” the test to automatically distinguish between pyramidal cells and interneurons, defining “ground truth” by the presence or absence of an apical dendrite. We compared hierarchical clustering with a battery of different supervised classification algorithms, finding that supervised classifications outperformed hierarchical clustering. In addition, the selection of subsets of distinguishing features enhanced the classification accuracy for both sets of algorithms. The analysis of selected variables indicates that dendritic features were most useful to distinguish pyramidal cells from interneurons when compared with somatic and axonal morphological variables. We conclude that supervised classification algorithms are better matched to the general problem of distinguishing neuronal cell types when some information on these cell groups, in our case being pyramidal or interneuron, is known *a priori*. As a spin-off of this methodological study, we provide several methods to automatically distinguish neocortical pyramidal cells from interneurons, based on their morphologies. © 2010 Wiley Periodicals, Inc. Develop Neurobiol 71: 71–82, 2011

## INTRODUCTION

**Figure 1:**
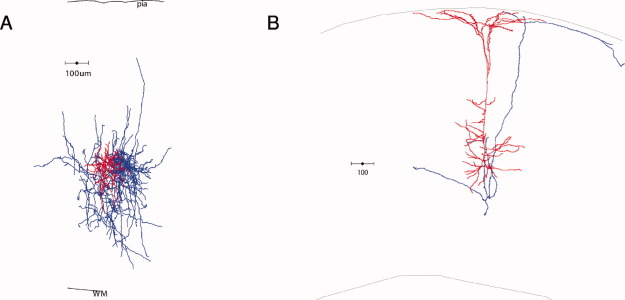
“Benchmark” task: distinguishing between GABAergic interneurons and pyramidal cells. Representative basket (A) and pyramidal (B) cell from mouse neocortex. Axonal arbor in blue and dendritic tree in red. Data examples obtained from http://www.columbia.edu/cu/biology/faculty/yuste/databases.html.

To understand neural circuits it is necessary, as a first step, to correctly identify the existing subtypes of neurons, before one tries to discern how they are connected and how the circuit functions. For neocortical circuits in particular, the two principal neuronal types of the cerebral cortex (see Fig. 1) are pyramidal cells and GABAergic interneurons (Ramón y Cajal,^1899^; Peters,^1987^). This basic classification has been expanded over the last century with the discovery of new subtypes of cells. At the same time, classification of cortical neurons has traditionally been qualitative (de Nó,^1922^) with nomenclature that varies across investigators. For this reason, it is apparent that a classification based on quantitative criteria is needed, in order to obtain an objective set of descriptors for each cell type that most investigators can agree upon. As suggested by community efforts (Ascoli et al.,^2008^) proper neuronal type definition should probably be a multimodal information task, including physiological, molecular and morphological features, and should use classification algorithms that are both quantitative and robust (Cauli et al.,^2000^).

Previous efforts to quantitatively classify cortical neurons have based their neuronal classification on unsupervised clustering techniques (Cauli et al.,^2000^; Kozloski et al.,^2001^; Wong et al.,^2002^; Tsiola et al.,^2003^; Benavides-Piccione et al.,^2005^; Dumitriu et al.,^2007^; Helmstaedter et al.,2008a,b,c; Karagiannis et al.,^2009^; McGarry et al.,^2010^). These are essentially exploratory techniques which aim at discovering new subtypes of cells or confirming some known hypothesis about them. But in these studies, prior information on the potential outcomes was not utilized, or was only used to validate the clustering. Instead, this information could be used to guide a supervised classification. An example of this approach can be seen in Marin et al. (2002), where linear discriminant analysis was used to investigate whether different classes of projection neurons have distinct axon projection patterns, a problem also tackled by Wong et al. (2002), using hiearchical clustering.

In our study, we compare the performance of supervised and unsupervised classification approaches in an apparently simple task: to automatically distinguish interneurons from pyramidal cells. It is important to note that, in this benchmark exercise, the presence or absence of an apical dendrite was not included in the morphological features, since it was used as the “ground truth” to evaluate the performance of the algorithms. More specifically, we compared hierarchical clustering using Ward's method, the most common unsupervised algorithm used with neuronal data, with different supervised algorithms such as naïve Bayes, C4.5, k-nn, multilayer perceptron and logistic regression. Supervised methods outperformed hierarchical clustering, confirming the power of adding additional statistical descriptors to the task. In addition, since the inclusion of all the available variables could potentially lead to a less accurate model, we explored whether selecting subsets of variables improved classification, for both supervised and unsupervised methods. We tested wrapper, embedding and filter selection methods, finding that they indeed significantly improve the classification using both types of algorithms.

## METHODS

### Preparing Brain Slices

All animal experiment was done in compliance with the IACUUC from Columbia University. Live brain slices were prepared from the cortex of PND 14 C57/B6 mice. Mice were decapitated using scissors. The skin and skull were removed. The brain was then immediately placed in cold sucrose artificial cerebral spinal fluid (222 m*M* sucrose, 2.6 m*M* KCl, 27 m*M* NaHCO_3_, 1.5 m*M* NaH_2_PO_4_, 2 m*M* CaCl_2_, 2 m*M* MgSO_4_, bubbled with 95% 02, 5%CO_2_) for 3 min. The brain was then transferred to a cutting block with the cortex facing up. Slices 300–400 μm thick were cut using a Vibratome. The slices remained viable for several hours for use in various electrophysiology experiments.

### Histological Procedure

Neurons were filled with biocytin by a patch pipette. Slices were kept overnight in 4% paraformaldehyde in 0.1 *M* phosphate buffer (PB) at 4°C. The slices were then rinsed three times for five minutes per rinse on a shaker in 0.1 *M* PB. They were placed in 30% sucrose mixture (30 g sucrose dissolved in 50 mL ddH20 and 50 mL 0.24 *M* PB per 100 mL) for 2 h and then frozen on dry ice in tissue freezing medium. The slices were kept overnight in a −80°C freezer. The slices were defrosted and the tissue freezing medium was removed by three 20-min rinses in 0.1 *M* PB while on a shaker. The slices were kept in 1% hydrogen peroxide in 0.1 *M* PB for 30 min on the shaker to pretreat the tissue. The slices were rinsed twice in 0.02 *M* potassium phosphate saline (KPBS) for 20 min on the shaker. The slices were then kept overnight on the shaker in Avidin-Biotin-Peroxidase Complex. The slices were then rinsed three times in 0.02 *M* KPBS for 20 min each on the shaker. Each slice was then placed in DAB (0.7 mg/mL 3,3″-diaminobenzidine, 0.2 mg/mL urea hydrogen peroxide, 0.06 *M* Tris buffer in 0.02 *M* KPBS) until the slice turned light brown and was then immediately transferred to 0.02 *M* KPBS and transferred again to fresh 0.02 *M* KPBS after a few minutes. Stained slices were then rinsed a final time in 0.02 *M* KPBS for 20 min on a shaker. Each slice was then mounted onto a slide using crystal mount.

### Reconstruction of Neuron Morphologies

Successfully filled and stained neurons were reconstructed using Neurolucida (MicroBrightField). Neurons were viewed with 60× oil objective on an Olympus IX71 inverted light microscope or an Olympus BX51 upright light microscope. For intricate sections of the neuron a 100× oil objective was used. The Neurolucida program projects the microscope image onto a computer drawing tablet. The user then traced the neuron's processes while the program recorded the coordinates of the tracing to create a three dimensional image. The user defined an initial reference point for each tracing. The *z* coordinate was then determined by adjustment of the focus. In addition to the neuron, the pia and white matter were drawn.

The Neurolucida Explorer program was used to measure sixty four morphological variables of the reconstruction as well as the relative distance of the soma to the pia. Some variables were directly measured, such as somatic area and perimeter, number of axons and dendrites, axonal and dendritic length, axonal and dendritic branch angles and number of axonal and dendritic nodes (branch points). Other variables were calculated values such as axon and dendritic Sholl lengths, convex hull analysis, and fractal analysis. Sholl length is a measure of how the length of the processes is distributed. Concentric spheres centered at the soma were drawn around the neuron; for axons the spheres were drawn at radius intervals of 100 μm and for dendrites at intervals of 50 μm. The Sholl length is the total length of the part of the axon or dendrite contained within in each shell. Convex hull analysis draws a convex shape around the axons or dendrites in both two (*x,y*) and three (*x,y,z*) dimensions. The area and perimeter of the two dimensional shape and the volume and surface area of the three dimensional shape are then calculated. Fractal analysis calculates the fractal dimension of the axons or dendrites using linear regression, and thus is a measure of how the neuron fills space.

### Unsupervised Classification

In unsupervised learning or clustering (Jardine and Sibson,^1968^), the aim is to discover groups of similar instances within the data. In this approach, we have no information about the class label of data or how many classes there are.

### Unsupervised Classification Algorithms

**Figure 2:**
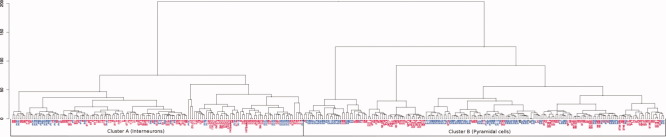
Graphical representation of a hierarchical clustering (dendrogram). Interneurons are labeled in red and pyramidal cells in blue. Note that although there are two major clusters which represent mostly interneurons and pyramidal cells, there are many of misplaced neurons in this type of unsupervised classification.

One of the most common unsupervised methods is hierarchical clustering, previously used to classify neurons (see Section 1). This is an approach based on organizing data into a hierarchical structure according to the proximity matrix. The graphical representation of the clustering is a tree structure, called dendrogram (see Fig. 2). In these methods, agglomerative clustering is usually used and works from the bottom up, by merging nearest clusters at each step. The merger depends on a measure of dissimilarity. Euclidean distance is normally used as a measure of distance between pairs of observations and Ward's method is the linkage criteria to specify the dissimilarity between clusters in our case.

The ultimate clustering results are obtained by slicing the dendrogram at a particular level. In our case, this level is when only two clusters remain, attempting to separate pyramidal cells in a cluster and interneurons in the other.

### Supervised Classification

We used different supervised classification algorithms. In addition, we assessed and compared the performance of these algorithms to determine if supervised classification outperformed unsupervised clustering and if so which algorithms were most effective.

### Supervised Classification Algorithms

In supervised classification, each instance is represented by a vector (***x***^(*j*)^, *c*^(*j*)^), with *j* ∈ {1, … , *N*}, where ***x***^(*j*)^ is composed by the values of n predictor variables or features and *c*^(*j*)^ denotes one of the *r*_0_ labels ∈ {1, … , *r*_0_} of the class variable *c*. The task is to automatically induce a model based on a set of *N* instances, called training data. This model then will be used to assign labels to new instances with unknown labels using only the value of their predictor variables. If we have a new instance ***x***, supervised classification builds a function γ such that:


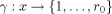
(1)

The chosen algorithms in this article are representative from several paradigms, because it is not known *a priori* which one is more suitable for this type of data. Next, a short description of each algorithm used is presented:

Naïve Bayes (NB) (Minsky,^1961^), derived from Bayesian classifiers. The maximum a posteriori assignment to the class label is based on obtaining the conditional probability density function for each feature given the value of the class variable.

C4.5 (Quinlan,^1993^), derived from classification trees. It builds a decision tree from the training data using recursive partitioning of the space representing the predictive variables and based on the information gain ratio.

K-nn (Cover and Hart,^1967^), derived from “lazy algorithms,” called K-nearest neighbors. It is based on classifying instances assigning labels guided by the K nearest instances labels. This algorithm does not provide an explicit model.

Multilayer perceptron (MLP) (Rumerlhart et al.,^1986^), derived from neural networks. This is an artificial neuronal network and is based on simulating the structure and behavior of the biological neuronal networks.

Logistic regression (LR) (Hosmer and Lemeshow,^2000^), derived from statistical theory. This algorithm builds a model estimating parameters using the maximum likelihood estimation method.

**Figure 3:**
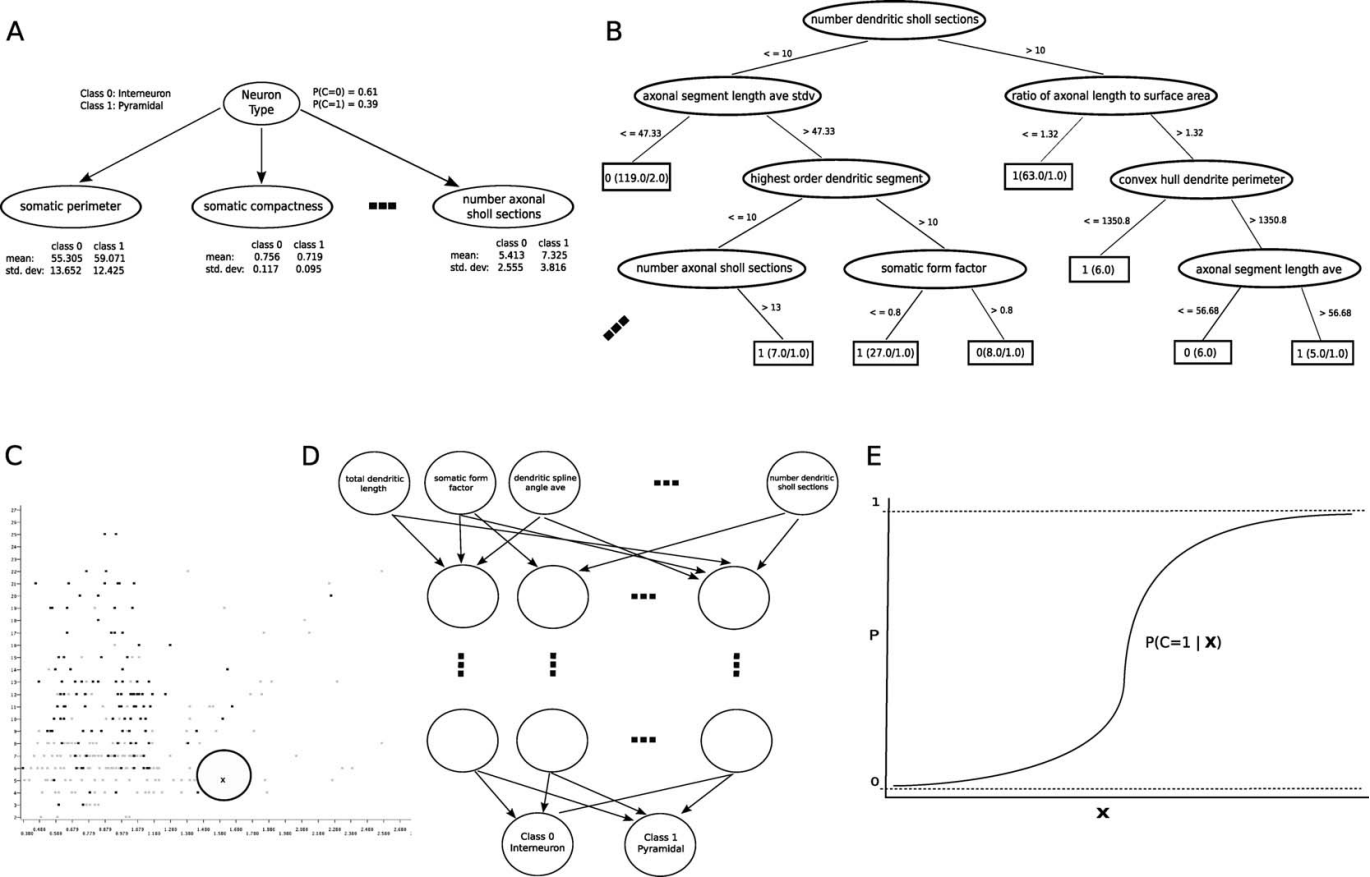
Example of the models obtained from the supervised classification algorithms used in this study. A: Partial naïve Bayes model. For each class label and feature, mean and standard deviation (std. dev.) are shown. B: Partial classification tree model obtained from C4.5 algorithm. C: Projection of data in 2D. In k-nn, each instance is classified based on the class label of its k nearest neighbors. This algorithm does not build a model. D: Partial multilayer perceptron model. A neural network is built with an input, output and several hidden layers. E: Graphical representation of a logistic function, base of the logistic regression model.

An example of the models built using these classification algorithms is shown in Figure 3.

### Assessing Classification Algorithms

The chosen measure of classification performance is the rate of correctly classified instances, using the presence or absence of an apical dendrite as the ground truth. To evaluate the performance of a model, the evaluation should be carried out on data not seen in training the model. One problem of using a completely different dataset to test and to train the model is that information in the test set could have significant information that is lost as it is never used to train the model.

One common technique that can evaluate performance without losing information is *k*-fold cross-validation (Stone,^1974^). The data set of size m is randomly partitioned into *k* sets (“folds”) all of size m/*k*. Then *k*-1 folds are used to train a model, which is then evaluated on the one unused fold. This process is repeated *k* times, each time leaving out a different fold for evaluating the model. The final performance measure of the model is the average of the *k* runs.

### Statistical Test to Compare Models

A 10-fold cross-validation was used to estimate the performance of each supervised classification algorithm, so there are 10 values of this performance for each algorithm. To correctly compare the performance of the different classification algorithms, these distributions of values must be compared using a statistical hypothesis test.

In our case, we used the Wilcoxon signed-rank test (Wilcoxon,^1945^). It is a nonparametric statistical hypothesis test which can reveal the existence of significant differences between two distributions. Our null hypothesis is that there are not statistical differences between the two distributions. The procedure for using the Wilcoxon signed-rank test was to compare the distribution obtained using the model with the highest averaged rate of correctly classified instances against each of the other distributions obtained with the rest of models.

### Dimensionality Reduction

To reduce the number of variables, we explored two strategies: feature extraction (PCA) and feature subset selection (FSS).

#### Principal Component Analysis

Principal component analysis (PCA) (Jolliffe,^1986^) is a very popular method for feature extraction. PCA obtains new uncorrelated variables named principal components (PCs), which preserve as much of the original information as possible. These principal components are sought from the original features and maximize the data variance captured. It is a mathematical procedure and can be calculated from the eigenvalue decomposition of the data covariance matrix.

#### Feature Subset Selection

We also used feature subset selection (FSS), a different method for dimensionality reduction based on selection instead of extraction. The rationale is that not all variables that are measured for data analysis are likely to be necessary for building an accurate model and including all of them may lead to a less accurate model than if some of them were removed. The problem is that it is not obvious *a priori* which variables are relevant and/or nonredundant. Besides, this dimensionality reduction can lead to more parsimonious, or easily understood, models. Other advantages could be the decrease in the cost of data acquisition or the faster induction of the model. For all these reasons, FSS was carried out in our study.

There are three approaches to perform FSS (Kohavi and John,^1997^; Liu and Motoda,^1998^): filter, which ranks the subsets of features based on intrinsic characteristics of the data independently of induction learning algorithms; wrapper, which evaluates the FSS with the accuracy of the learning algorithm; and embedded, where FSS is part of the process itself in some learning algorithms such as C4.5. In addition, three searching techniques were used to seek in the space of predictor variables when it is necessary in filter and wrapper approaches: forward selection, backward elimination (Kittler,^1978^), and genetic algorithms (Goldberg,^1989^). Genetic algorithm search procedure evolves good feature subsets by using random perturbations of a current list of candidate subsets. Each individual of the genetic algorithms is a binary string of size *n* (total number of features) and represents the selected features.

To perform the classification and the FSS, Weka software (Witten and Frank,^2005^) was used with all the parameters by default. Specifically, for the genetic algorithm, default parameters as number of generations and probabilities of crossover and mutation were those implemented in Weka. The population individuals were chosen at random. The statistical tests, the PCA analysis and hierarchical clustering were run using the statistical package R (Ihaka and Gentleman,^1996^).

## RESULTS

Our goal was to compare the performance of hierarchical clustering and supervised classification algorithms in the benchmark task of distinguishing between pyramidal cells and interneurons, based solely on their morphological differences. We used a database of 327 cells (199 interneurons and 128 pyramidal cells), and for each cell, 65 morphological features were measured, creating a data matrix (Supporting Information Table 1). All pyramidal neurons had clear apical dendrites. Interneurons belonged to many different subtypes and were collected over several different studies from the laboratory. For each algorithm the exercise consisted in optimally classifying all neurons into two groups: pyramidal cells or interneurons. We assessed the percentage of correctly classified cells by taking into account which neurons had or lack an apical dendrite, information which was not used by the unsupervised algorithms, and was only used by the supervised algorithms during the training phase.

### Clustering Algorithms

We first performed clustering using hierarchical Ward's method, the most common classification algorithm used with neuronal data. This approach was used with three different dimensionality reduction techniques. The first one was based on the first six principal components (PCs) obtained with PCA, which carry almost 55% of the total variance. This number of PCs was chosen because of the trade-off between the accuracy and the number of features. For example, using the first seven PCs (60% of the total variance), the accuracy decreased by 2%. Using the first eleven PCs (70% of the total variance), the accuracy was only increased in 1%. And finally, using the first 16 PCs (80% of the total variance), the accuracy decreased in 4%. The second variable selection method for clustering was to use only those original features with a correlation coefficient greater than 0.7 with the first six PCs. With this requirement, 10 original features remained. Finally, filter FSS was used as the third method to select variables in unsupervised approach.

As we knew beforehand which neurons were pyramidal and which were interneurons, the accuracy of the hierarchical clustering was calculated as the percentage of each group of cells which fall in the correct majority cluster, after separating the data into two final clusters. Thus, we assumed that each cluster was equivalent to a class.

All the hierarchical clustering results can be seen in Table 1. Without dimensionality reduction techniques, 59.33% of accuracy was obtained. Using the above techniques of dimensionality reduction related to PCA the outcomes were relatively poor. Only 59.02% accuracy was reached using PCA, which is the lowest value from all algorithms in this comparative study. Using hierarchical clustering of the more than 0.7 correlated features with the PCs, the accuracy obtained was 66.77%. This is increased when the features obtained with filter FSS were used. The accuracy obtained is 71.25% using backward elimination, and this value increased to 77.68% using forward selection and 79.82% using genetic algorithms.

**Table 1:** Results Obtained with Hierarchical Clustering Using Ward's Method

		Hierarchical Clustering	
		
		Accuracy	#
No FSS		59.33	65
PCA	PC	59.02	6
	Original features	66.77	10
Filter	Forward	77.68	10
	Backward	71.25	17
	Genetic	79.82	16

PC uses the six first principal components, whereas “Original Features” uses the original features with correlation greater than 0.7 with the six first principal components. The number of features used (#) is also indicated.

As mentioned, all these accuracy values were obtained without using any previous information about the class variable. Supervised classification algorithms, whose results are presented next, use this known information to build the different models.

### Supervised Classification Algorithms

A battery of different supervised classification algorithms, listed in the Methods section, were compared in the task of distinguishing between pyramidal cells and interneurons. Again, we first used all the available data, without FSS. Filter FSS was then used with three different search strategies, the same as with hierarchical clustering. Finally, we explored wrapper FSS, another approach used to select subsets of features (see Methods section) which is only appropriate for supervised classification algorithms. Thus, a comparison using it with clustering techniques cannot be made.

### Naïve Bayes

This algorithm obtained very similar results using all variables and using variables selected by the filter FSS process (see Table 2). Without FSS, an 80.73% ± 10.44% accuracy was achieved, whereas with filter FSS, the accuracy was around 80%. Wrapper FSS was able to improve these means: with forward search, its accuracy was 87.16% ± 6.34%. Backward (83.18% ± 9.12%) and genetic search (83.49% ± 8.55%) did not significantly improve the accuracy.

**Table 2:** Results Obtained Using Naïve Bayes (NB)

		NB	
		
		Accuracy	#
No FSS		80.73 ± 10.44	65
Filter	Forward	79.82 ± 9.86	10
	Backward	79.51 ± 9.74	17
	Genetic	80.43 ± 7.07	16
Wrapper	Forward	**87.16** ± **6.34**	**8**
	Backward	83.18 ± 9.12	50
	Genetic	83.49 ± 8.55	23

Values correspond to the accuracy of each model, i.e. the mean ± standard deviation (percentage) averaged over the 10 values estimated using 10-fold cross-validation. The number of features used (#) is also indicated as before. Bold face indicates the model with no significant statistical differences with the highest accuracy supervised model.

### C4.5

In the case of C4.5 algorithm, all the results (see Table 3) were comparable or better than those obtained using naïve Bayes. Without FSS, an 84.40% ± 3.84% of accuracy was obtained. Forward selection and genetic algorithms for filter FSS showed lower outcomes than without FSS, but by using backward selection a performance of 88.07% ± 6.09% using only 11 features was achieved. This mean was the highest one obtained using filter FSS. In the case of wrapper FSS, the outcomes were 86.85% ± 5.29% using forward selection, 87.16% ± 5.83% using backward selection and 86.85% ± 4.72% using genetic search.

**Table 3:** Results Obtained Using the Decision Tree C4.5

		C4.5	
		
		Accuracy	#
No FSS		84.40 ± 3.84	65
Filter	Forward	82.26 ± 7.17	9
	Backward	**88.07** ± **6.09**	**11**
	Genetic	81.65 ± 7.24	6
Wrapper	Forward	86.85 ± 5.29	7
	Backward	**87.16** ± **5.83**	**12**
	Genetic	**86.85** ± **4.72**	**13**

### K-nn

K-nn was configured with *k* = 5 after trying some preliminary tests, this configuration obtained better accuracy than *k* = 1, *k* = 3, and *k* = 7. In spite of 5-nn being the simplest algorithm used to classify, the results (see Table 4) were quite competitive with other approaches. Specifically, with 5-nn using all the available variables a 83.18% ± 7.15% accuracy is obtained. This value improved when filter FSS is used, obtaining 85.01% ± 5.60% with genetic algorithms as the best case. Again, wrapper FSS was the best approach to select appropriate variables, with accuracies using backward selection of 86.85% ± 6.26%, and in turn, this is overcome by 87.46% ± 5.68% for genetic algorithms and 89.30% ± 7.58% for forward selection.

**Table 4:** Results Obtained Using *K*-nn (with *K* = 5)

		5-nn	
		
		Accuracy	#
No FSS		83.18 ± 7.15	65
Filter	Forward	83.79 ± 9.55	10
	Backward	84.71 ± 6.03	17
	Genetic	85.01 ± 5.60	16
Wrapper	Forward	**89.30** ± **7.58**	**6**
	Backward	86.85 ± 6.26	51
	Genetic	**87.46** ± **5.68**	**34**

### Multilayer Perceptron

Multilayer perceptron (see Table 5) was the algorithm with the highest overall accuracy among all the algorithms without using FSS (87.46% ± 9.06%). Moreover, this result was improved using backward selection for filter FSS (87.77% ± 6.36%). However, using forward selection (82.57 ± 9.54) or genetic algorithms (82.26% ± 9.17%), the accuracy was reduced. The improvement obtained using wrapper FSS was not as significant as when using other supervised algorithms. In this case, 88.07% was the highest accuracy mean obtained using forward selection (±4.99) and backward elimination (±8.27).

**Table 5:** Results Obtained Using Multilayer Perceptron (MLP)

		MLP	
		
		Accuracy	#
No FSS		**87.46** ± **9.06**	**65**
Filter	Forward	82.57 ± 9.54	10
	Backward	87.77 ± 6.36	17
	Genetic	82.26 ± 9.17	16
Wrapper	Forward	**88.07** ± **4.99**	**10**
	Backward	**88.07** ± **8.27**	**61**
	Genetic	87.46 ± 6.26	37

### Logistic Regression

The last supervised classification algorithm, logistic regression (see Table 6), maintained the mean obtained without FSS (82.26% ± 7.36%) when forward selection for filter FSS was used (82.26 ± 9.82). This outcome is 83.49 ± 9.45 using genetic algorithms while using backward elimination reaches 85.63% ± 8.56%. The highest accuracy of all the approaches was obtained using logistic regression with wrapper FSS and a genetic algorithms search: 91.13% ± 5.95%. This model was therefore used in the statistical test (see Methods section) to be compared against the other models.

**Table 6:** Results Obtained Using Logistic Regression (LR)

		LR	
		
		Accuracy	#
No FSS		82.26 ± 7.36	65
Filter	Forward	82.26 ± 9.82	10
	Backward	85.63 ± 8.56	17
	Genetic	83.49 ± 9.45	16
Wrapper	Forward	**85.63** ± **9.79**	**7**
	Backward	84.71 ± 7.54	59
	Genetic	**91.13** ± **5.95**	***33***

Details as before.

### Feature Selection and Comparison Between Clustering Approach and Supervised Classification

For hierarchical clustering, filter FSS always generated more accurate classifications than using all available variables, or after applying some traditional dimensionality reduction technique such as PCA. It is important to highlight this result because all previous clustering work uses PCA to reduce the number of variables. Specifically, for our benchmark test, using filter FSS enhanced accuracy of unsupervised clustering by almost 15%. Thus, this approach appears desirable to select an appropriate subset of variables for future cluster analysis studies.

When comparing hierarchical and supervised methods, we find that hierarchical clustering and filter FSS, using forward selection or genetic algorithms, were competitive combinations against supervised classification algorithms with no FSS and filter FSS. On the other hand, when wrapper FSS is used with the supervised classification algorithms it is generally superior.

### Comparison Among Supervised Classification Algorithms

After concluding that supervised methods with wrapper selection of variables enhance the classification, the next step was to determine which supervised algorithm was best able to discriminate between pyramidal cells and interneurons in our benchmark test.

The highest accuracy was obtained using the model built with logistic regression and wrapper FSS (with a genetic algorithm). To compare this model with all the rest, the Wilcoxon signed-rank test was used.

The results obtained with this statistical test are shown in Table 7. In this table, only the models which have a *p*-value greater than 0.05 (differences are not statistically significant) in the test are shown. As these models did not reject the null hypothesis, we cannot assert than they are significantly different from the model built using logistic regression and genetic algorithms in a wrapper approach. Thus these models are the top models from our results. Statistical hypothesis test outcomes confirm that models obtained with the wrapper approach are the most accurate to classify interneurons and pyramidal cells, since nine of the selected models in Table 7 are built using wrapper FSS.

**Table 7:** Results of Wilcoxon Signed-Rank Test

FSS		Algorithm	*p*-Value
No FSS		MLP	0.091
Filter	Backward	C4.5	0.095
Wrapper	Forward	NB	0.095
		5-nn	0.220
		MLP	0.063
		LR	0.053
	Backward	C4.5	0.077
		MLP	0.115
		C4.5	0.052
	Genetic	5-nn	0.052
		LR	–

Models which do not reject the null hypothesis, and therefore, with no significant statistical differences (*p*-value greater than 0.05) with the highest accuracy model are listed.

These results indicate that there is not one particular supervised method which is superior, since all the used algorithms could be chosen as winners based on the statistical test. Therefore, an appropriate selection of variables (using wrapper FSS in our case) appears to be more important than using a specific supervised algorithm.

### Features That Distinguish Between Interneurons and Pyramidal Cells

We finally explored which of the morphological features, or combinations of them, were most indicative of differences between pyramidal cells and interneurons. In the original data set, 65 variables were available before applying subset selection. When filter FSS was applied, the number of attributes obtained for each searching method was the same, except for C4.5 algorithm. This is because filter FSS algorithms do not depend on the classification method to obtain the subset of features. The number of features selected, using filter FSS, was 10 for forward selection, 17 for backward selection, and 16 for genetic algorithms. C4.5 is the only algorithm with different number of features, since it has an embedded FSS that chooses a subset from the features selected by the filter FSS to build the decision tree.

The number of features selected using wrapper FSS were similar but the main difference was in the searching technique. Using forward selection, the number of features selected was in a range from 6 to 10. The low number of features is a bias of the forward selection. In the case of backward elimination, the number of features was higher (from 50 to 61) with an exception in the C4.5 algorithm. In C4.5, the number of features selected by the wrapper FSS was 23, and after that, when C4.5 induces the decision tree model, only 12 features were used. Genetic algorithms technique selects from 13 to 37 features, taking into account again that C4.5 has the embedded FSS. This technique was not as biased as the two others, since it is not a “greedy” search.

Regarding the specific features chosen, somatic compactness seemed to be the most important somatic feature because it was the most commonly selected variable by the winner models. As for axonal features, the number of axonal Sholl sections and standard deviation of the average axonal segment length were the two most important features. This can be seen in the logistic regression and C4.5 models for example, because these two features had a high coefficient or are located at the top of the tree [see Fig. 3(B)]. In addition, the axonal local angle average was another important feature because it was selected by many models. For the same reasons, the number of dendritic Sholl sections and the ratio of dendritic length to surface area were the most important dendritic features. The highest order dendritic segment is selected by the majority of the models as well.

We also performed tests using separately the somatic, axonal and dendritic subsets of features on some of the selected models (unpublished results). While models built using only somatic features obtained ∼60% accuracy, ∼75% accuracy was obtained with axonal features while dendritic features reached ∼85% accuracy (not shown). These values confirmed the importance of dendritic features.

Therefore, our results indicate that dendritic features are very informative to differentiate morphologically pyramidal neurons from interneurons, although some axonal and somatic features also contribute to this distinction.

## DISCUSSION

To enable the quantitative classification of neuronal cell types, in this methodological study we have compared different methods to distinguish between neuronal classes, based on their morphologies. By using a standard database with a clearly classified set of cells, we devised a benchmark test in which the algorithms had to distinguish pyramidal cells from interneurons. A human observer originally classified these cells into both classes according to the presence or absence of an apical dendrite, thus setting the ground truth for this task. We then tested side by side the performance of the unsupervised clustering method, which is becoming standard in neuroscience, versus the performance of representative algorithms from some of the most popular supervised classification methods used in machine learning. Our reason for doing so is that, if previous information is available to classify data, taking advantage of it to obtain more accurate outcomes should be desirable. Nevertheless, given the peculiarities of the classification problem, it was not obvious that that supervised methods world be in principle better than previously used neuronal classifiers, or which approach could outperform the others, so we undertook the task of carefully comparing a battery of algorithms and different preprocessing strategies.

Our main finding is that supervised classification methods outperformed unsupervised algorithms. In this comparative study, we show that hierarchical clustering approach is unable to obtain accuracy as precise as supervised classification when distinguishing between pyramidal cells and interneurons. Therefore, supervised classification is an effective approach to perform this task and is another approach in neuronal data analysis, which that could be useful in future studies. In fact, previous classification studies, in which some information is known beforehand, could be reanalyzed using that information as a class label with supervised algorithms. An ideal supervised classification algorithm does not emerge from our results. It seems that the accuracy of results obtained does not depend on the classification algorithm, since the best models chosen using the statistical test are built using all the different supervised classification algorithms tested. Thus, the choice of the algorithm would depend on each specific classification or domain. There could be some bias in this choice, since if an interpretable model is desirable C4.5 or naïve Bayes could be the most preferred. 5-nn does not build a model, so this could be an undesirable restriction.

Our second conclusion is that the preselection of the variables with FSS greatly enhances the performance of both supervised and unsupervised methods. Specifically, in terms of which FSS approach to follow, we find that the wrapper FSS is the most suitable technique for our data set of neurons using supervised algorithms. Models obtained using FSS are desirable, not only because a higher accuracy is achieved, but also because more parsimonious and easily understood models are obtained. The disadvantage of this approach is its computational cost, since performing wrapper FSS is slow. Wrapper FSS cannot be used with unsupervised algorithms, but the results obtained using a different variable preselection method, the filter FSS, with hierarchical clustering point out the advantage of using this dimensionality reduction technique, compared to clustering with no FSS.

Our final conclusion is that an acceptable distinction between interneuron and pyramidal cells was achieved using dendritic morphological features, even without explicitly providing knowledge of the presence or absence of an apical dendrite.

### Future Directions

Our work establishes, for the first time to our knowledge, the use of several supervised methods for classifying and distinguishing between neuronal cell types. While differentiating between pyramidal neurons and interneurons may not seem a particularly difficult task for a trained neuroanatomist, distinguishing subtypes of neurons using objective and quantitative criteria is more challenging. Therefore, we expect that the supervised classification methods that we introduce here, which are standard in machine learning, could help future neuroscience research, particularly with respect to classifying subtypes of neurons. For example, one future direction could be the quantitative exploration of new subtypes of interneurons. For this goal, unsupervised clustering techniques could still be used as exploratory techniques. However, supervised classification could greatly help to obtain more accurate classifications when information on class labels is known beforehand and an accurate FSS or a reliable validation could be obtained as well.

An ultimate, more ambitious, goal could be to arrive at an objective classification of all neuronal cell types, based on their morphologies or on a combination of morphological, physiological, and molecular criteria (Ascoli et al.,^2008^). To accomplish this goal, *a priori* information will probably be most useful, or even key. For this task, one could explore the use of semisupervised clustering, using previous information about known cell groups that are very homogeneous or represent a single cell type, for example chandelier cells in neocortex, as a way to partially supervise the clustering. Although it is difficult to reach a consensus about the known cell types that exist in the cortex, the introduction of supervised, or partially supervised algorithms could help advance the state of this key question, which is essential to decipher neocortical circuits.
